# Social interactions and COVID-19 vaccine hesitancy: Evidence from a full population study in Sweden

**DOI:** 10.1371/journal.pone.0289309

**Published:** 2023-11-20

**Authors:** Johan Klaesson, José Lobo, Charlotta Mellander

**Affiliations:** 1 Jönköping International Business School, Jönköping University, Jönköping, Sweden; 2 Institute of Retail Economics (HFI), Stockholm, Sweden; 3 Arizona State University, Tempe, AZ, United States of America; Singapore General Hospital, SINGAPORE

## Abstract

We investigate whether an individual’s information milieu―an individual’s residential neighborhood and co-workers―affects the decision to get a COVID-19 vaccine. The decision to accept or refuse a vaccine is intensely personal and involves the processing of information about phenomena likely to be unfamiliar to most individuals. One can thus expect an interplay between an individual’s level of education and skills and the information processing of others whom with whom she can interact and whose decision she can probe and observe. Using individual-level data for adults in Sweden, we can identify the proportion of an individual’s neighborhood and workplace who are unvaccinated as indicators of possible peer effects. We find that individuals with low levels of educational attainment and occupational skills are more likely to be unvaccinated when exposed to other unvaccinated individuals at work and in the residential neighborhood. The peer effects in each of these information milieus further increases the likelihood of not getting vaccinated—with the two acting as information channels that reinforce one another.

## Introduction

Even before the current global pandemic, the World Health Organization (WHO) and the European Centre for Disease Prevention and Control declared vaccine hesitancy― the choice to delay or refuse available vaccination―as a major challenge to public health care policy worldwide [1, 2]. Although the rapid development of several vaccines against COVID-19 was welcomed by the majority of affected communities, widespread COVID-19 vaccine hesitancy was equally a defining characteristic of this epidemiological episode [3–6]. The motivations driving this hesitancy have received a lot of attention and include a wide range of possible factors: public distrust in government agencies; inadequate publicity and educational campaigns; concerns about the side effects of the various vaccines; receipt of disinformation or inaccurate information about the dangers and consequences of COVID-19 infection; demographic, socioeconomic and ethnic characteristics (age, gender, income, employment status); educational level; concerns about infringement on civil liberties by vaccination mandates; religious beliefs; prior concerns about vaccines; skepticism about the severity or even reality of the pandemic; and negative sentiments in social media [7–14].

The decision to accept or refuse to be vaccinated is intensely personal yet has community-wide implications. It involves weighing the risks, evaluating the costs and benefits, and assessing the medical, immunological, and biological information. The decision-making process may be influenced by many factors, including one’s educational and cultural background; social and political forces; vaccine-specific concerns [15–20]; and even the groups and communities in which an individual is embedded [16, 21]. Education and skill level are also expected to strongly correlate with health literacy skills, empowering individuals to access, comprehend, and utilize information to promote and maintain good health, particularly in complex healthcare programs such as vaccination, where critical analysis of information is crucial [22]. In addition, the earliest vaccines for the SARS-CoV-2 virus were developed in an unprecedented fast timeframe, and because they functioned biologically different than “standard” vaccines [23], they complicated the decision for some to get vaccinated.

The ability and necessity of humans to learn from each other has long been recognized as a defining feature of Homo sapiens [24]. Such learning can take many forms. Role modeling and peer group influences generate imitative behavior, a type of information processing whereby the behavior of others affects an individual’s evaluation of the consequences of their own choices [24]. “Neighborhood effects”—the effects on individual level outcomes based on the behavior of members of an individual’s residential community—have also long been a phenomenon of interest in urban sociology [25–28]. In particular, the effects of a neighborhood’s characteristics on the health of its residents have received attention [29–32]. While economics has typically studied aggregate behavior as the outcome of individual decisions made interactively, and sociology has focused on the role of social influences on individual behavior, researchers now recognize that when making decisions with significant economic consequences, individuals can be directly influenced by the choices and characteristics of others around them. This effect creates an information feedback loop between individual and group choices based on the past choices of some people, which then affect the current social context and hence future choices of others [33, 34]. Given this, information provided by friends, relatives, acquaintances, neighbors and workmates, along with the diversity of views provided in one’s social networks and the scale and density of the social networks in which an individual is embedded can all complement an individual’s own knowledge when making decisions [35–37].

Thus, when needing to make decisions under the constraints of limited knowledge, resources, and time, and in settings characterized by risk and uncertainty, reliance on group behavior can be an effective heuristic [38].

That humans learn from, influence, and are influenced by others, should therefore be expected to sway an individual’s decision to get vaccinated or not. More specifically, the likelihood that an individual gets vaccinated might indeed be affected by the prevalence of vaccination rates in that individual’s neighborhood and workplace. But as we commonly see in the study of social interactions, observable outcomes may be generated by many different interaction processes, so empirical findings are open to a variety of confounding effects and interpretations [39].

In this work, we ask:

1. Are individuals with lower education and/or skill levels less likely to get the COVID-19 vaccination, controlling for many other characteristics?
2. Are individuals with lower education and/or skill levels more likely to be influenced by peers (neighbors) in the residential area where they live and/or by peers (colleagues) at work in their decision to get the COVID-19 vaccination?

To be able to answer these research questions, data is needed that makes it possible to link an individual’s decisions with the specific characteristics and behaviors of those with whom she interacts and who constitute her neighborhood and social groups. This is an empirical tall order for most population studies, but detailed Swedish micro-level data allows us to both measure salient characteristics of an individual, including whether the individual received a COVID-19 vaccine, and who constitute her residential and workplace peers.

## Data and methods

### Modelling the determinants of vaccination hesitancy

We consider the individual’s attitude towards vaccination as being an unobservable (latent) variable that can be interpreted as the individual’s perceived net benefit from getting a vaccine shot. The net benefit is defined as the sum of all (psychic) benefits minus the sum of all (psychic) costs of getting the vaccine shot. Included is all available information that goes into the decision-making process regarding whether to get the shot or not. Choice factors may include the risk of getting COVID-19 (and potentially becoming very sick or even dying), the perceived benefit or danger of the vaccination itself, and the logistical costs of getting the vaccine (e.g., taking time off from work, incurred transportation cost, etc.). The net benefit cannot be observed and is thus labeled a latent variable.

We can, however, observe the final decision made between getting the vaccination or not. We assume that individuals make this choice by weighing the pros and cons of vaccination against COVID-19. The assumed decision rule that people follow is that if the benefits outweigh the costs, they get the shot. If the costs are higher than the benefits, they refrain from getting the shot.

To investigate the role of education and skills in relation to the likelihood of not getting the COVID-19 vaccine, we utilize a binary Logit regression analysis. The Logit model is often used to model discrete choice outcomes and is thus appropriate to use in this case since we want to model the likelihood of an event occurring or not. The event we are interested in is the occurrence of an individual choosing *not* to get the vaccine shot against COVID-19. The alternative is to get the shot, so the choice is binary in nature. In the analysis, we code not taking the shot as a one and taking the shot as a zero.

The above-described decision rule can be formalized as in Eq (1), where we consider the perceived net benefit of getting the vaccine as a function of measurable explanatory variables. Eqs (2) and (3) model the discrete choices of whether to get the vaccine shot or not, based on its perceived net benefit being positive or negative.

$$y_{i}^{*}=x_{i}\beta_{i}+e_{i}$$ (1)

$$y_{i}=1\;if\;y_{i}^{*}<0$$ (2)

$$y_{i}=0\;if\;y_{i}^{*}\geq 0$$ (3)

y^*^_i_ is individual i’s net benefit from getting the vaccine shot. *x*_*i*_ is a vector of explanatory variables and *β_i_* are the corresponding coefficients to be estimated. *y*_*i*_ corresponds to the action we observe, i.e. the individual either got the shot or did not.

We assume that the probabilities of the outcomes (0 or 1) follow the logistic cumulative distribution function. This amounts to assuming that the probability (*Pr*) of the outcome (not taking the vaccine) follows an s-shaped (sigmoid) curve for increasing values of the explanatory variables where the probability goes from zero to one. The so-called logit is defined as the logarithm of the odds of the modeled outcome and is expressed as:

$$\ln\left(\frac{Pr}{1-Pr}\right)$$

Estimating the logit as a linear function of explanatory variables (*x_i_β_i_*) gives us Eq (4):

$$Pr(y_{i}=1|x_{i})=\frac{1}{1+exp(-x_{i}\beta_{i})}$$ (4)

which is the ordinary binomial logit model that we will use to estimate the probability of observing individuals choosing not to get the vaccine. Higher values of *x*_*i*_ will give higher probabilities of the outcome “individual i chooses not to get vaccinated,” given that the coefficient *β_i_* is a positive number. In the empirical estimations we estimate the value of the coefficients *β_i_*.

### Data

To study the role of education and skill level, and the role of peers on the likelihood of not getting vaccinated, we employ individual level register data for the full Swedish population aged 16 years and above from Statistics Sweden (MONA). The data covers 8,665,827 individuals and includes information about age, educational degree, income, occupation, civil status, housing type, workplace ID, as well as residential neighborhood location and electoral district. Based on this, we are able to identify who an individual works with as well as who the individual’s neighbors are. The *neighborhood* spatial unit used is “Regional Statistical Area” (RegSO) which is constructed by Statistics Sweden to facilitate measuring socio-economic structures. This definition of neighborhood takes factors such as household income, type of housing, demographic homogeneity, and location-specific demographic conditions into account. Neighborhoods are defined by spatial borders, for example, streets, waterways, and railways. In other words, these neighborhood areas represent actual neighborhoods, not administrative constructs.

The *workplace* is defined based on the establishment (i.e., physical place of work) where an individual works, not the firm. This means that we are able to identify with whom an individual interacts at work, since every individual in the register data with the same workplace ID will be employed by the same firm in the same physical space.

We then use data from the National Vaccination Register (NVR) for the time period December 27^th^, 2020, to May 4^th^, 2022. The register, introduced in 2013, is administrated by the Public Health Agency of Sweden (Swe: Folkhälsomyndigheten). By law, it is obligatory for all health providers to report all vaccinations covered by the national vaccination program to the register. Every COVID-19 vaccination that took place during this time is registered at the individual level. Other vaccinations besides COVID-19 that are reported to the register include diphtheria, tetanus, whooping cough, polio, haemophiles, influenzae type b, pneumococcus, measles, mumps, rubella, human papillomavirus, and rotavirus. Since the law did not come into effect until 2013, we do not have information about whether individuals in the data may have gotten other types of vaccinations earlier in life.

By merging the two datasets—Statistics Sweden and the National Vaccination Register—we obtain a unique dataset with information about the full Swedish population aged 16 and above with detailed socio-economic characteristics as well as whether an individual had a registered COVID-19 vaccination (information about ethics approval and other information about the data can be found at the end of this document).

Approximately 12.5 percent of individuals registered with Statistics Sweden are not registered in the National Vaccination Register, indicating they did not have the COVID-19 vaccination. It is also possible that some of these individuals got vaccinated in another country, which would not be noted in the National Vaccination Register. However, given that travel was restricted during our study´s time period, we believe that the vast majority of the 12.5 percent not-registered group consciously decided not to get vaccinated.

As stated, based on the register data from Statistics Sweden, we have access to the ID of an individual’s workplace and geo-coded residential information. Based on these data, we are able to calculate the unvaccinated shares in both the workplaces and neighborhoods, which in turn provides us with information about with whom each individual potentially interacts on a daily basis. In our dataset, 5,572,609 individuals have a registered occupation. The primary reason for not having a registered occupation is because the individual is retired, but it includes as well those who are unemployed or studying. When we calculate the unvaccinated shares in workplaces, we only include workplaces with five employees or more to avoid getting high shares driven by only one or a few individuals. This further reduces our data to 4,625,719 individuals, and this becomes the dataset upon which we run our regression estimations.

Based on this dataset, we investigate the influence of educational attainment, occupational skill, and the possible role of peers in an individual’s neighborhood and workplace for the likelihood of deciding not to get the COVID-19 vaccine shot. A person is considered vaccinated if she/he got at least one shot of a COVID-19 vaccine. We estimate this probability based on several individual and peer level variables.

We use a logit regression analysis as per our motivation above. We estimate the relationship between educational attainment and occupational skill as explanatory variables for the probability of not getting COVID-19 vaccinated (the dependent variable). We also include information about whether the individual’s peers at work and in the neighborhoods got COVID-19 vaccinated. It is important to note that we employ data for the relevant full population. This means that the results obtained are not dependent on any prior sampling. To answer our research questions, we formulate the regression setup as follows:

P(unvaccinated)=f(education,occupation,otherindividualcharacteristics,peersinneighborhood,peersatwork)

*P* indicates probability and *f* denotes the function outlined in Eq (4) above and the different variables in the paratheses corresponds to the vector *x*_*i*_ in Eq (4). A detailed description of all the included variables is available in the S1 File.

## Results

### Educational attainment, occupational skill, and vaccination rates

Table 1 presents the COVID-19 unvaccinated shares of individuals by educational attainment and occupation type, presented in order of length of education (short to long) and expected occupational skill level (high to low). Occupational skill in this context refers to the knowledge and abilities expected of those who perform specific job tasks, depending on the degree of autonomy and complexity of the job.

**Table 1:** Share of COVID-19 unvaccinated by educational attainment and occupational skill group.

**Education Level**	**Population shares not vaccinated**
9 years or less	15.3%
Vocational High School	11.5%
High School 2 years (Higher Education Preparatory)	14.7%
High School 3 years (Higher Education Preparatory)	12.5%
Higher Education less than 3 years	10.1%
Higher Education 3 to 4 years	7.4%
Higher Education 5 years or more	9.4%
PhD	5.4%
N = 8,358,914	
**Occupation**	**Population shares not vaccinated**
Managers	4.4%
Occupations requiring advanced level of higher education	5.6%
Occupations requiring higher education qualifications or equivalent	7.6%
Administration and customer service clerks	11.5%
Service, care, and shop sales workers	13.1%
Agricultural, horticultural, forestry and fishery workers	9.6%
Building and manufacturing workers	16.3%
Mechanical manufacturing and transport workers, etc.	15.5%
Elementary occupations	15.0%
N = 5,572,609	

Unvaccinated shares tend to decrease with the length of education. Among those who have 9 years of education or less, equivalent to having finished junior high school, 15.3% are not vaccinated. This is nearly three times the rate of those who hold a PhD (5.4%). There is also a significant difference between the high- and low-skill occupational groups. The occupational groups are defined by Statistics Sweden and build on The International Classification of Occupations (ISCO). Managers, the occupational group with the highest degree of autonomy and complexity, as well as “occupations requiring advanced levels of education,” have unvaccinated shares ranging from 4.4 to 7.6%. This rate is less than half of those in building and manufacturing, mechanical manufacturing and transportation, and elementary occupations (15.0 to 16.3%).

Next, we run a logit regression, where we take other individual-level characteristics such as age, gender, ethnicity, marital status, and housing situation into account. The average marginal effects from the estimation are illustrated in Table 2 with delta-method standard errors within parentheses. Average marginal effects are a useful way to present the results from a logit regression since the probability for each individual will differ in a logit estimation. The coefficients should therefore be interpreted as the average change in probability of not getting COVID-19 vaccinated when the variable changes by one unit expressed as percentage points. It is important to recall that the average marginal effects that we estimate do not imply that we test for causality.

**Table 2:** Logit regression results of the likelihood of not getting vaccinated (average marginal effects).

***Education***	
Baseline: 9 years or less	
Vocational High School	-0.005
	(0.0005)
High School 2 years (Higher Education Preparatory)	-0.015
	(0.0007)
High School 3 years (Higher Education Preparatory)	-0.021
	(0.0006)
Higher Education less than 3 years	-0.029
	(0.0005)
Higher Education 3 to 4 years	-0.040
	(0.0006)
Higher Education 5 years or more	-0.049
	(0.0008)
PhD	-0.044
	(0.0015)
***Occupation***	
Baseline: Managers	
Occupations requiring advanced level of higher education	0.015
	(0.0006)
Occupations requiring higher education qualifications or equivalent	0.022
	(0.0007)
Administration and customer service clerks	0.032
	(0.0008)
Service, care and shop sales workers	0.031
	(0.0007)
Agricultural, horticultural, forestry and fishery workers	0.037
	(0.0016)
Building and manufacturing workers	0.045
	(0.0008)
Mechanical manufacturing and transport workers, etc.	0.052
	(0.0008)
Elementary occupations	0.029
	(0.0008)
***Peer Impact Controls***	
Unvaccinated share in neighborhood	-0.470
	(0.0037)
Unvaccinated share in workplace	-0.310
	(0.0025)
Interaction Unvaccinated share in neighborhood and workplace	3.442
	(0.0015)
**Control for individual characteristics (e.g., age, income, civil status, foreign-born, housing type, voting results in the election district)**	**YES**
**Control for Municipality**	**YES**
N	4,625,719
Pseudo R2	0.183

Based on the regression analysis, we still find that the relationship between education and the avoidance of the COVID-19 vaccination is negative. Individuals with a higher education were approximately 2.9 (a coefficient equal to -0.029) to 4.9 (-0.049) percentage points less likely to be unvaccinated than the baseline group (those with an education of 9 years or less). Similarly, the likelihood of not getting vaccinated increases with lower occupational skill levels. Individuals in administration and customer service; service, care, and shop sales; agricultural, horticultural, forestry, and fishery; or mechanical manufacturing and transportation were 3.7 (0.037) to 5.2 (0.052) percentage units less likely to be vaccinated than the baseline group (managers). Taken together, the results suggest that low-educated, low-skilled individuals are more likely to be vaccine hesitant, even once other factors have been controlled for.

The negative sign for the variables *unvaccinated share in neighborhood* and *unvaccinated share in workplace* is a result of including the interaction variable between these two variables. When the interaction variable is excluded, the association with the unvaccinated shares in neighborhoods and workplace is positive (as per S2 Table, Model 1 and 4 in the S1 File). Also, when the interaction variable is included, the total effect becomes positive due to the size of coefficients in combination with the possible range of values taken by the explanatory variables.

For robustness checks, we run alternative versions of the logit regression (S2 Tabe in S1 File). First, we re-run the estimations for individuals in workplaces with two instead of five or more employees (S2 Table, Model 2 in S1 File). This increases the number of observations from 4,625,719 to 4,960,899. We also re-run the full regression without the workplace variable (which is presented in the S1 File). Here, we include individuals who do not have a workplace because of, as noted above, either retirement, studying, or unemployment (Model 3, n = 8,665,827). Last, we re-run the logit regression but exclude the interaction variable (Model 4, n = 4,625719). The results for our main variables of interest are robust with these variations in specification.

### Unvaccinated peers

Table 3 illustrates the vaccination shares for individuals, neighborhoods, and workplaces depending on educational attainment and occupational skill level. Not only are individuals with lower levels of education and skills less often vaccinated against COVID-19, but we also see differences in vaccination rates in these individuals’ neighborhoods and workplaces (i.e., where they live and work).

**Table 3:** Share of COVID-19 unvaccinated by neighborhood and workplace.

	Individual Level	By neighborhood	By workplace
**High Education**	9.4%	11.5%	7.9%
**Low Education**	13.7%	11.9%	10.4%
**High Skill**	7.4%	10.6%	6.7%
**Low Skill**	15.1%	12.4%	11.2%

The widest differences are found at the individual level, but there is also a significant difference in vaccination shares at the workplace level. This illustrates how individuals with low levels of education and skills tend to be around unvaccinated people on a daily basis while working. In contrast, there is only a minor difference in non-vaccination rates at the neighborhood level based on educational attainment, but a bigger difference when we differentiate groups based on skill level.

### The role of peer-groups

Our second question asked: How do peer groups with low vaccination rates at the workplace and in the neighborhood correlate with the probability of an individual not getting vaccinated? To answer this, we use a probability surface graph, which contains information about the probability of an event given a certain data point. The height of the surface over the zero-plane shows the expected probability. The surface shows how the combination of unvaccinated shares in the residential neighborhood and workplace influences the likelihood that an individual does not get a COVID-19 vaccine. The x-axis indicates the share of unvaccinated workplace colleagues. The z-axis indicates the share of unvaccinated individuals in the neighborhood, while the y-axis indicates the probability that an individual does not get a COVID-19 vaccine. The probability surface is calculated based on estimated parameters using the logit model.

**Fig 1:**
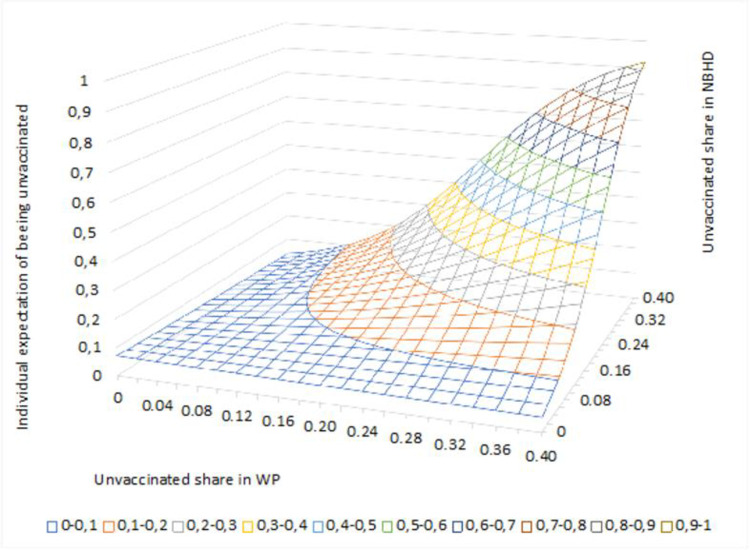
Probability surface of the individual expectation of being unvaccinated in relation to the unvaccinated shares in the workplace and the neighborhood.

The graph in Fig 1 illustrates how individuals who live and work in places with lower shares of unvaccinated individuals (bottom left) are less likely to be unvaccinated (blue area). This area covers most individuals since the majority live and work in places where a relatively low share of their peers is unvaccinated. It is also worth noting that increasing either the share of unvaccinated people in the neighborhood or in the workplace alone does not necessarily increase the risk of not getting vaccinated, but when the shares increase in both places in combination, the risk increases (orange area).

Next, we divide the population of individuals into groups based on their educational attainment (two groups) and occupational skill level (two groups) and re-estimate the potential influence of unvaccinated peers in the residential neighborhood and workplace. Thus, the estimations are performed using four different groups. We recall from Table 3 that individuals with high levels of educational attainment and occupational skills, on average tend to live in neighborhoods and work in workplaces with lower shares of unvaccinated neighbors and colleagues.

**Fig 2:**
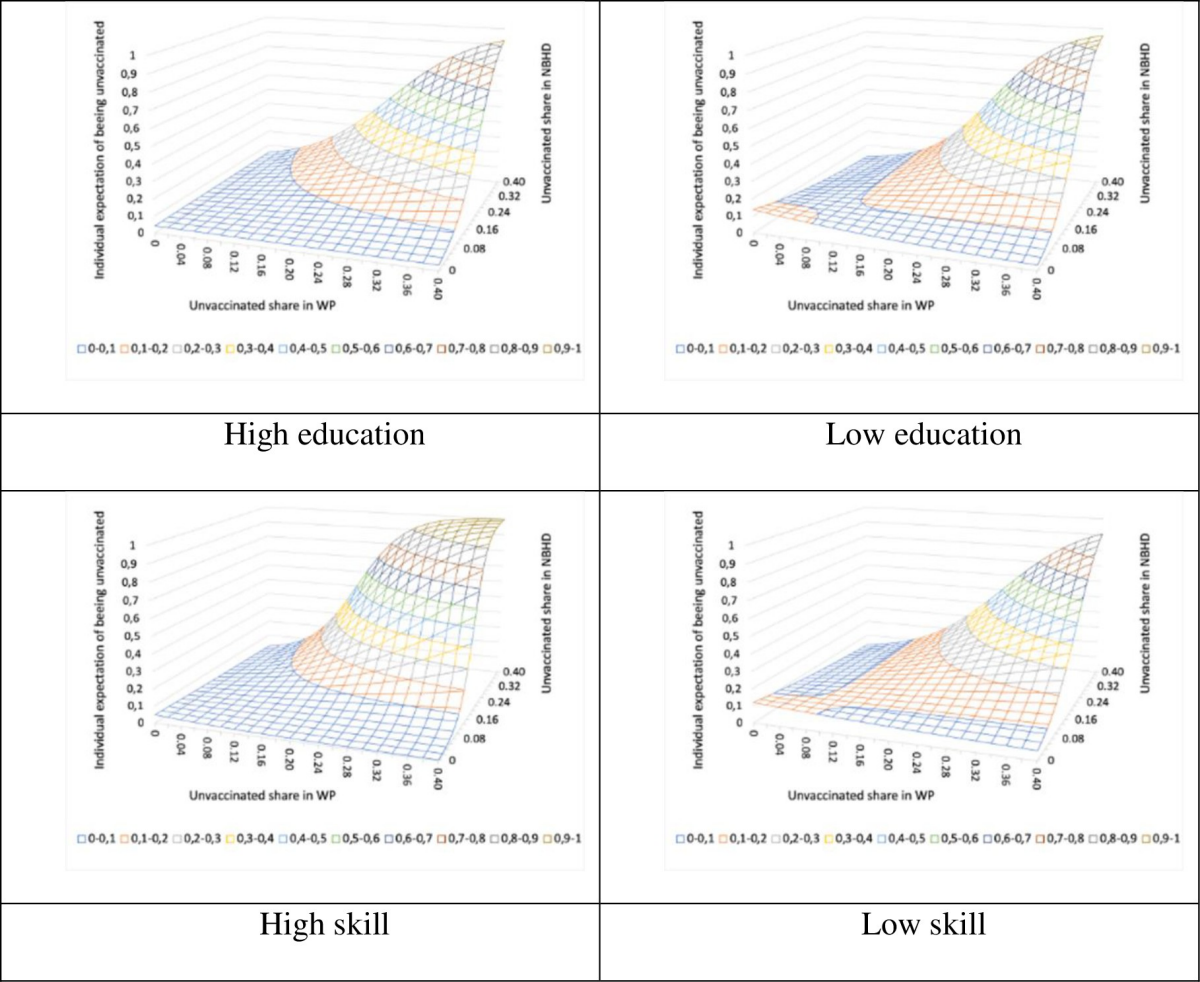
Probability surfaces of the individual expectation of being unvaccinated in relation to the unvaccinated shares in the workplace and the neighborhood (Different education levels and skill levels).

Fig 2 illustrates how the probability surface of the individual probability of being vaccinated changes based on educational attainment and occupational skill level. The probability surfaces are obtained using the estimated regression parameters for each of the four groups. We find that the blue area, which represents a very low probability of not getting the vaccine, increases with high education and skill levels. In other words, the probability of being influenced by unvaccinated peers tends to be relatively low, even in combination. In comparison, individuals with lower education and skill levels are at a higher risk of not being vaccinated especially when the unvaccinated shares increase in both their neighborhood and workplace. These results indicate that low-educated and low-skilled individuals are more likely to be unvaccinated than highly educated and skilled individuals when they live and work in places with more unvaccinated individuals.

## Discussion

The starting premise for the investigation is that the decision to get vaccinated requires the processing of information about phenomena likely to be unfamiliar to most individuals. Besides a reliance on official recommendations, individuals will rely on the information processing of others with whom they interact and whose decisions they observe. The results presented here strongly suggest that an individual’s information milieu, specifically the residential neighborhood as well as co-workers at the same workplace location, influences the decision to get vaccinated or not. Both the educated and highly skilled and the relatively uneducated and low-skilled individuals will note what their peers do. For Swedish adults, our findings indicate that low levels of education and skills could affect the choice to get vaccinated against COVID-19. The results also suggest that peers (neighbors and co-workers) of the low-educated and low-skilled individuals―who themselves tend to have low levels of education and skills―might reinforce such vaccine hesitation.

To the best of our knowledge, there has not been a prior study that comprehensively examines vaccine hesitancy using micro-level data as we have done, including information about individual decision-making regarding the COVID-19 vaccine, their socioeconomic status, and the characteristics of the communities in which they are embedded. Our study stands out as unique in this regard. However, our findings are in line with a study conducted in southern Sweden, which revealed that individuals living in areas with lower socioeconomic status were less likely to receive the COVID-19 vaccine [16]. Our results also support previous research that emphasizes the significant impact of individual-level factors, such as age, gender, ethnic background, income, and civil status, on vaccine hesitancy [14, 16–19].

Despite Sweden’s small-size and its political and cultural peculiarities as a Scandinavian society long-governed by social democratic precepts, the findings presented here should be of relevance to many other nations, even those that are comparably less developed and wealthy. The “information neighborhood effects” posited in this paper with respect to the decision to receive the COVID-19 vaccine likely occurs in any society, whatever its level of socioeconomic development. As in the case of Sweden, vaccine hesitancy appears to be closely intertwined with an individual’s educational attainment, neighborhood of residence, and workplace. For individuals with low educational attainment, what neighborhood and workplace peers do or do not do regarding vaccination can significantly inform their vaccination choice.

As with every study, ours comes with some limitations. A primary one is that we cannot control for individuals’ information sources among their social media networks, which may play a role in the decision to be vaccinated. An individual may also belong to other informal networks that we cannot control for, e.g., religious groups, where the leaders may play a role in the spread of information about vaccinations–especially locally in certain ethnic groups. Our findings should, of course, be considered in light of such caveats.

In sum, the decision to get vaccinated is not merely the consequence of an individual-level cost-benefit assessment performed in isolation, but is one influenced by peer-effects. Recognizing this has valuable implications for future public vaccination campaigns, where information and educational efforts might need to be more strongly local in nature, reaching into neighborhoods and workplaces. The prevalence of vaccinated neighbors and workmates could be as influential as any government’s or health agency’s information and educational outreach activities themselves.

## Supporting information

S1 File Supplementary material is available including: Detailed information about variables, supplementary logit regressions, definition about country groups and supplementary figures. (DOCX)
