# Supplementary material for: Social interactions and COVID-19 vaccine hesitancy: Evidence from a full population study in Sweden
Source: PLoS One. 2023 Nov 20;18(11):e0289309. doi: 10.1371/journal.pone.0289309 (PMC10659190; doi:10.1371/journal.pone.0289309)
Supplement: S1 File — (DOCX) [file pone.0289309.s001.docx]

**Supplementary Material: Social Interactions and COVID-19 Vaccine Hesitancy: Evidence from a Full Population Study in Sweden**

S1: Variables Description.

| **Variable** | **Description** | | | |
| --- | --- | --- | --- | --- |
| Vaccinated | Binary variable equal to 1 if individual *i* got at least one vaccine shot against COVID-19, zero otherwise. Dependent variable in the selection equation. | | | |
| *Educational Group* | 9 Years or Less  Vocational High School  High School 2 years (Higher Education Preparatory)  High School 3 years (Higher Education Preparatory)  Higher Education less than 3 years  Higher Education 3 to 4 years  Higher Education 5 years or more  PhD | | | |
| Occupational Group | Managers  Occupations requiring advanced level of higher education  Occupations requiring higher education qualifications or equivalent  Administration and customer service clerks  Service, care and shop sales workers  Agricultural, horticultural, forestry and fishery workers  Building and manufacturing workers  Mechanical manufacturing and transport workers, etc.  Elementary occupations | | | |
| Unvaccinated share in neighborhood | The share of the population in the RegSO (neighborhood) aged 16 and above that did not get the COVID-19 vaccine. | | | |
| Unvaccinated share in the workplace | The share of the employees (N>=5) in the workplace that did not get the COVID-19 vaccine. | | | |
| Extreme right-wing voting in election district | The share of the voters in the election district that voted for the Sweden Democrates. | | | |
| *Individual Characteristics Control Variables* |  | | | |
| Ethnic background | Categorical variable (see Table S3 for included countries in each category). | | | |
|  | - Sweden (base)  - Nordic  - EU15  - West Balkan  - East Europe  - Middle East | | - East Africa  - North-South-West Africa  - South-Central Asia  - Southeast-East Asia  - South-Central America  - North America-Oceania^[[1]](#footnote-1)^ | |
| Income | The natural logarithm of disposable income, inferred from the whole family’s disposable income (disposable income per consumption weight). | | | |
| Historic Sick Leave | The number of sick leave days from 2015-2019. In Sweden, the first 14 days are paid by the employer. This is the number of days paid by the state, beyond these 14 days. | | | |
| Age | Categorical variable of age groups. | | | |
|  | - 15 – 29 (base)  - 30 – 39  - 40 – 49  - 50 – 59 | - 60 – 69  - 70 – 79  - 80 – 89  - 90 + | | |
| Female | Binary variable equal to 1 if female. | | | |
| Civil status | Categorical variable.  - Single with no children (base)  - Single with at least one child below 18  - Married/co-habitant with no children  - Married/co-habitant with at least one child below 18 | | | |
| Income | The natural logarithm of disposable income, inferred from the whole family’s disposable income (disposable income per consumption weight). | | | |
| Ethnic background | Categorical variable (see Table B1 for included countries in each category). | | | |
|  | - Sweden (base)  - Nordic  - EU15  - West Balkan  - East Europe  - Middle East | | | - East Africa  - North-South-West Africa  - South-Central Asia  - Southeast-East Asia  - South-Central America  - North America-Oceania^[[2]](#footnote-2)^ |
| Elderly home | Binary variable equal to 1 if residing in an elderly home. | | | |
| House type | Categorical variable.  - Rental apartment (base)  - Tenant-owned apartment  - Owner-occupied house | | | |
| Inter-generational household | Binary variable equal to 1 if residing in a household with at least one member aged below 18 and at least one member aged 70 or above. | | | |
| Crowded household | Binary variable equal to 1 if residing in a household classified as crowded, excluding single households without children in one-room apartments^[[3]](#footnote-3)^. | | | |

**S2: Full logit regression results for the likelihood of not getting vaccinated (average marginal effects).**

|  | **Model 1** | **Model 2** | **Model 3** | **Model 4** |
| --- | --- | --- | --- | --- |
| ***Education*** |  |  |  |  |
| Baseline: 9 years or less |  |  |  |  |
| Vocational High School | -0.005***  (0.0005) | -0. 004***  (0.0005) | -0. 005***  (0.0003) | -0.006***  (0.0005) |
| High School 2 years (Higher Education Preparatory) | -0.015***  (0.0007) | -0. 015***  (0.0007) | -0. 007***  (0.0005) | -0.012***  (0.0007) |
| High School 3 years (Higher Education Preparatory) | -0.021***  (0.0006) | -0.0020***  (0.0005) | -0.0015***  (0.0004) | -0.022***  (0.0006) |
| Higher Education less than 3 years | -0.029***  (0.0005) | -0. 027***  (0.0005) | -0. 028***  (0.0004) | -0.030***  (0.0005) |
| Higher Education 3 to 4 years | -0.040***  (0.0006) | -0.038***  (0.0005) | -0.038***  (0.0004) | -0.041***  (0.0006) |
| Higher Education 5 years or more | -0.049***  (0.0008) | -0.046***  (0.0008) | -0.038***  (0.0007) | -0.050***  (0.0008) |
| PhD | -0.044***  (0.0015) | -0.042***  (0.0015) | -0.029***  (0.0014) | -0.044***  (0.0016) |
| ***Occupation*** |  |  |  |  |
| Baseline: Managers |  |  |  |  |
| Occupations requiring advanced level of higher education | 0.015***  (0.0006) | 0.015***  (0.0006) | 0.005***  (0.0006) | 0.016***  (0.0007) |
| Occupations requiring higher education qualifications or equivalent | 0.022***  (0.0007) | 0.021***  (0.0007) | 0.017***  (0.0006) | 0.023***  (0.0007) |
| Administration and customer service clerks | 0.032***  (0.0008) | 0.032***  (0.0007) | 0.033***  (0.0007) | 0.030***  (0.0008) |
| Service, care and shop sales workers | 0.031***  (0.0007) | 0.031***  (0.0006) | 0.035***  (0.0006) | 0.030***  (0.0007) |
| Agricultural, horticultural, forestry and fishery workers | 0.037***  (0.0016) | 0.029***  (0.0014) | 0.044***  (0.0011) | 0.037***  (0.0017) |
| Building and manufacturing workers | 0.045***  (0.0008) | 0.041***  (0.0007) | 0.053***  (0.0007) | 0.045***  (0.0008) |
| Mechanical manufacturing and transport workers, etc. | 0.052***  (0.0008) | 0.051***  (0.0008) | 0.053***  (0.0007) | 0.049***  (0.0008) |
| Elementary occupations | 0.029***  (0.0008) | 0.028***  (0.0007) | 0.047***  (0.0007) | 0.030***  (0.0008) |
| ***Peer Impact*** |  |  |  |  |
| Unvaccinated share in neighborhood | -0.470***  (0.0037) | -0.656***  (0.0034) | 0.251***  (0.0017) | 0.208***  (0.0023) |
| Unvaccinated share in workplace | -0.310***  (0.0025) | -0.416***  (0.0021) | - | 0.241***  (0.0011) |
| Interaction Unvaccinated share in neighborhood and workplace | 3.442***  (0.0146) | 4.246***  (0.0125) | - | - |
| Extreme right-wing voting in election district | 0.002**  (0. 00003) | 0.002***  (0.00003) | 0.002***  (0.00002) | 0.002***  (0.00003) |
|  |  |  |  |  |
| ***Individual Level Control Variables*** |  |  |  |  |
| *Age* |  |  |  |  |
| 30 – 39 | 0.001***  (0.0004) | 0.002***  (0.0004) | 0.014***  (0.0004) | 0.003***  (0.0004) |
| 40 – 49 | -0.031***  (0. 0004) | -0.031***  (0. 0004) | -0.023***  (0. 0004) | -0.029***  (0. 0004) |
| 50 – 59 | -0.053***  (0. 0004) | -0.053***  (0. 0004) | -0.054***  (0. 0004) | -0.053***  (0. 0004) |
| 60 – 69 | -0.071***  (0. 0005) | -0.072***  (0. 0004) | -0.083***  (0. 0004) | -0.072***  (0. 0005) |
| 70 – 79 | -0.088***  (0. 0008) | -0.088***  (0. 0007) | -0.115***  (0. 0003) | -0.090***  (0. 0008) |
| 80 – 89 | -0.094***  (0. 0023) | -0.092***  (0. 0021) | -0.117***  (0. 0004) | -0.095***  (0. 0023) |
| 90 + | -0.061***  (0. 0016) | -0.055***  (0. 0014) | -0.096***  (0. 0007) | -0.062***  (0. 0017) |
|  |  |  |  |  |
| Female | -0.008***  (0.0003) | -0.006***  (0.0003) | -0.013***  (0.0002) | -0.008***  (0.0003) |
|  |  |  |  |  |
| *Civil status* |  |  |  |  |
| Single with child | -0.002***  (0. 0006) | -0.002***  (0. 0006) | -0.011***  (0. 0004) | -0.002***  (0. 0006) |
| Married | -0.021***  (0. 0004) | -0.023***  (0. 0004) | -0.028***  (0. 0004) | -0.020***  (0. 0004) |
| Married with child | -0.030***  (0. 0003) | -0.030***  (0. 0003) | -0.038***  (0. 0003) | -0.028***  (0. 0004) |
|  |  |  |  |  |
| Income | -0.025***  (0.0002) | -0.022***  (0.0002) | -0.026***  (0.0001) | -0.025***  (0.0002) |
| Historic Sick Leave | -0.0026***  (0.0011) | -0.0016***  (0.0010) | -0.0004***  (0.0005) | -0.0050***  (0.0011) |
|  |  |  |  |  |
| *Ethnic background* |  |  |  |  |
| Nordic | 0.035***  (0. 0012) | 0.035***  (0. 0012) | 0.043***  (0. 0008) | 0.033***  (0. 0012) |
| EU15 | 0.085***  (0. 0013) | 0.084***  (0. 0013) | 0.088***  (0. 0009) | 0.083***  (0. 0013) |
| West Balkan | 0.117***  (0. 0013) | 0.116***  (0. 0013) | 0.132***  (0. 0010) | 0.115***  (0. 0012) |
| East Europe | 0.178***  (0. 0012) | 0.172***  (0. 0011) | 0.215***  (0. 0009) | 0.176***  (0. 0011) |
| Middle East | 0.053***  (0. 0007) | 0.048***  (0. 0007) | 0.060***  (0. 0005) | 0.054***  (0. 0007) |
| East Africa | 0.044***  (0. 0011) | 0.047***  (0. 0011) | 0.037***  (0. 0007) | 0.035***  (0. 0010) |
| North-South-West Africa | 0.062***  (0. 0015) | 0.062***  (0. 0015) | 0.068***  (0. 0011) | 0.060***  (0. 0014) |
| South-Central Asia | -0.009***  (0. 0007) | -0.009***  (0. 0007) | -0.014***  (0. 0005) | -0.013***  (0. 0006) |
| Southeast-East Asia | -0.020***  (0. 0008) | -0.020***  (0. 0008) | -0.015***  (0. 0006) | -0.025***  (0. 0008) |
| South-Central America | 0.022***  (0. 0012) | 0.023***  (0. 0012) | 0.031***  (0. 0010) | 0.019***  (0. 0012) |
| North America-Oceania | 0.070***  (0. 0029) | 0.068***  (0. 0027) | 0.080***  (0. 0021) | 0.066***  (0. 0029) |
|  |  |  |  |  |
| Elderly home | -0.004  (0. 0040) | -0.002***  (0. 0039) | -0.031***  (0. 0011) | -0.004  (0. 0040) |
| *House type* |  |  |  |  |
| Tenant-owned (apartment) | -0.017***  (0. 0004) | -0.017***  (0. 0004) | -0.021***  (0. 0003) | -0.016***  (0. 0004) |
| Owner-occupied (house) | -0.018***  (0. 0004) | -0.019***  (0. 0004) | -0.019***  (0. 0003) | -0.015***  (0. 0004) |
| Inter-generational household | 0.023***  (0. 0017) | 0.023***  (0. 0016) | 0.047***  (0. 0012) | 0.022***  (0. 0017) |
| Crowded household | 0.021***  (0. 0004) | 0.021***  (0. 0004) | 0.033***  (0. 0004) | 0.022***  (0. 0004) |
|  |  |  |  |  |
| **Control for Municipality** | **YES** | **YES** | **YES** | **YES** |
| N | 4,625,719 | 4,960,899 | 8,665,827 | 4,625,719 |
| Pseudo R2 | 0.183 | 0.211 | 0.173 | 0.160 |

**S3: Countries included in each region of origin.**

| **Region of origin** | **Countries** |
| --- | --- |
| Sweden | Sweden |
| Nordic | Denmark, Finland, Norway, Iceland |
| EU15 | Austria, Belgium, France, Germany, Greece, Ireland, Italy, Luxembourg, Netherlands, Portugal, Spain, United Kingdom |
| West Balkan | Albania, Bosnia-Hercegovina, Kosovo, Macedonia, Montenegro, Yugoslavia |
| East Europe | Andorra, Bulgaria, Croatia, Cyprus, Czech Republic, Estonia, Hungary, Latvia, Liechtenstein, Lithuania, Malta, Moldavia, Monaco, Poland, Romania, Russia, San Marino, Serbia, Slovakia, Slovenia, Switzerland, Vatican City State |
| Middle East | Armenia, Azerbaijan, Bahrain, Georgia, ‎Iraq, Israel, Jordan, Kuwait, Lebanon,‎ Oman, Palestine, Qatar‎, Saudi Arabia, Syria, Turkey, United Arab Emirates, Yemen |
| East Africa | Burundi, Comoros, Djibouti, Eritrea, Ethiopia, Kenya, Madagascar, Malawi, Mauritius, Mozambique, Rwanda, Seychelles, Somalia, Tanzania, Uganda, Zambia, Zanzibar, Zimbabwe |
| North-South-West Africa | All other countries in Africa |
| South-Central Asia | Afghanistan, Bangladesh, Bhutan, India, Iran, Kazakhstan, Kyrgyzstan, Maldives, Nepal, Pakistan, Sikkim, Sri Lanka, Tajikistan, Turkmenistan, Uzbekistan |
| Southeast-East Asia | Brunei, Burma (Myanmar), Cambodia, China, Hong Kong, Indonesia, Japan, Laos, Malaysia, Mongolia, North Korea, the Philippines, Singapore, South Korea, Taiwan, Thailand, Timor-Leste, Vietnam |
| South-Central America | All countries in South and Central America |
| North America-Oceania | All countries in North America and Oceania |

**S4: Figures for workplaces with 2 employees or more**


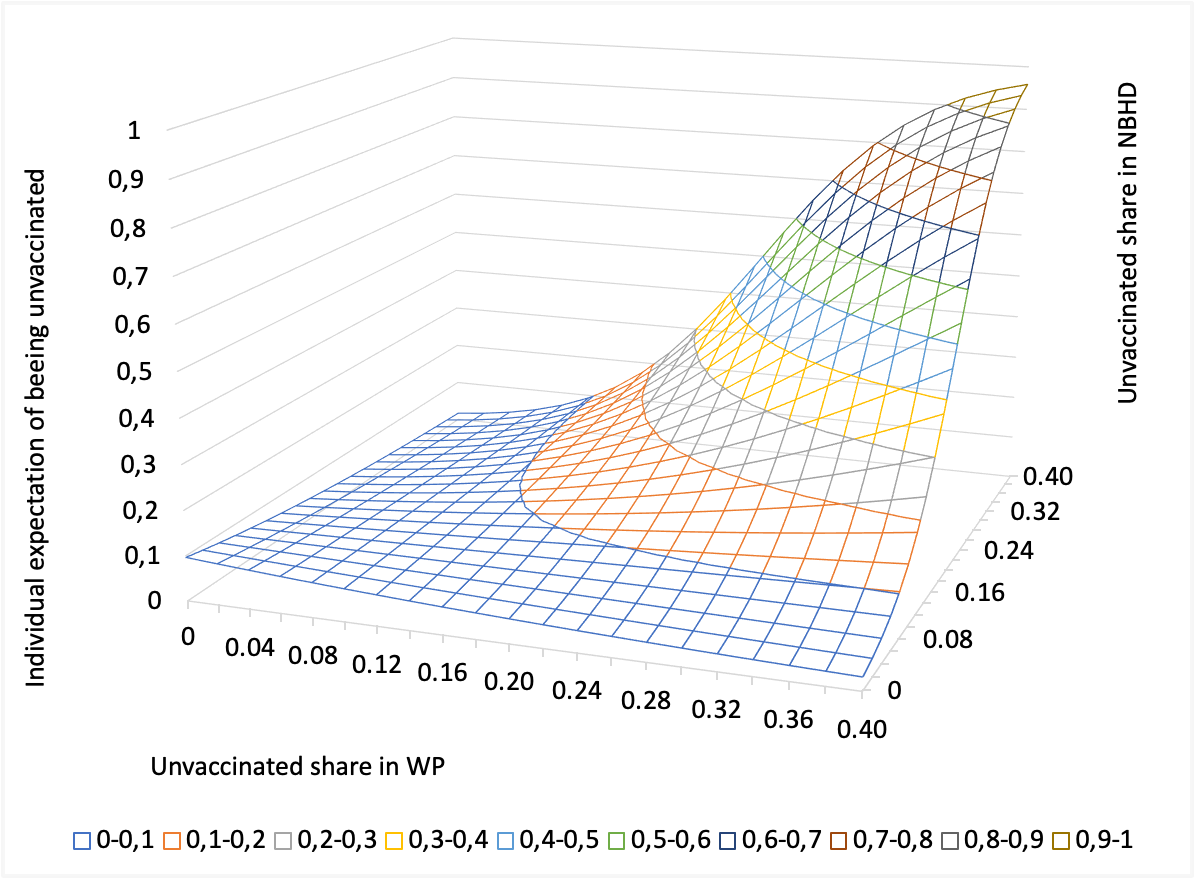


**Probability surface of the individual expectation of being unvaccinated in relation to the unvaccinated shares in the workplace and the neighborhood**

| 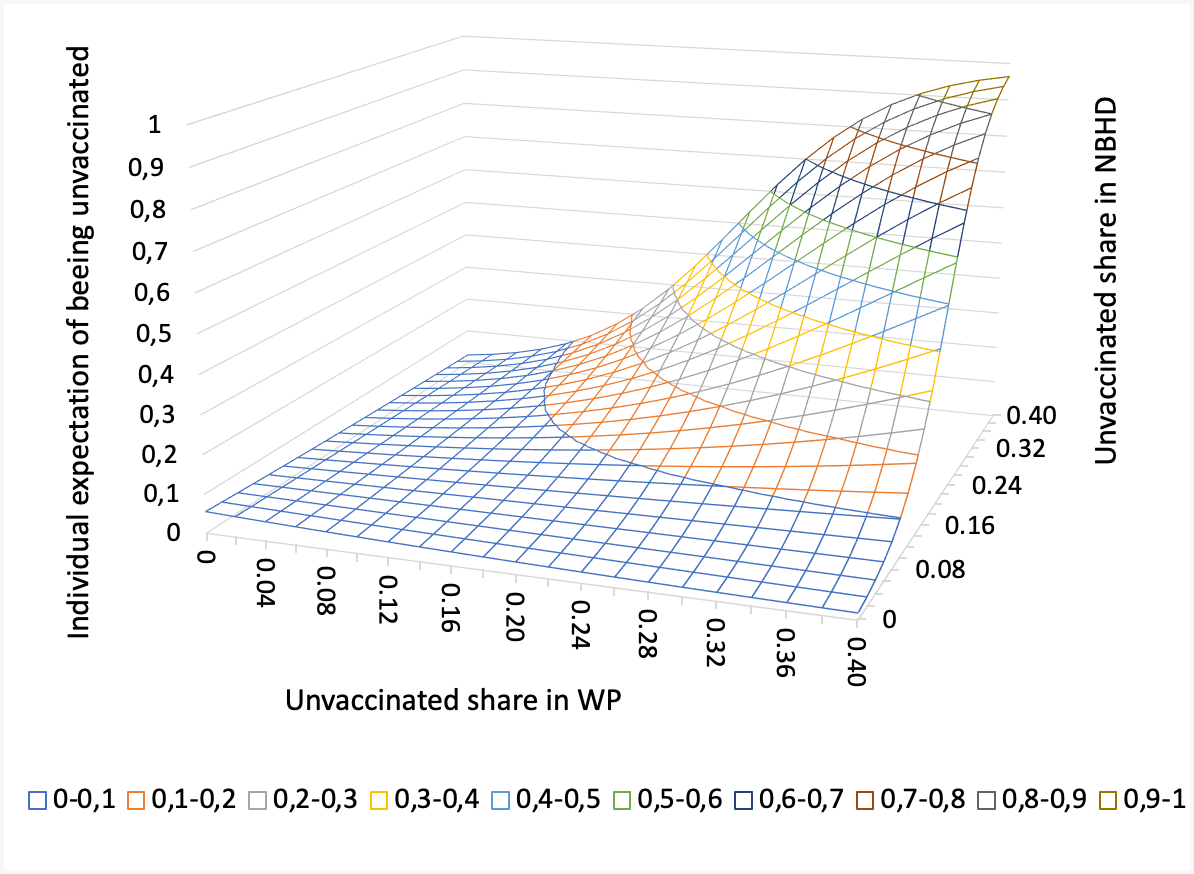 | 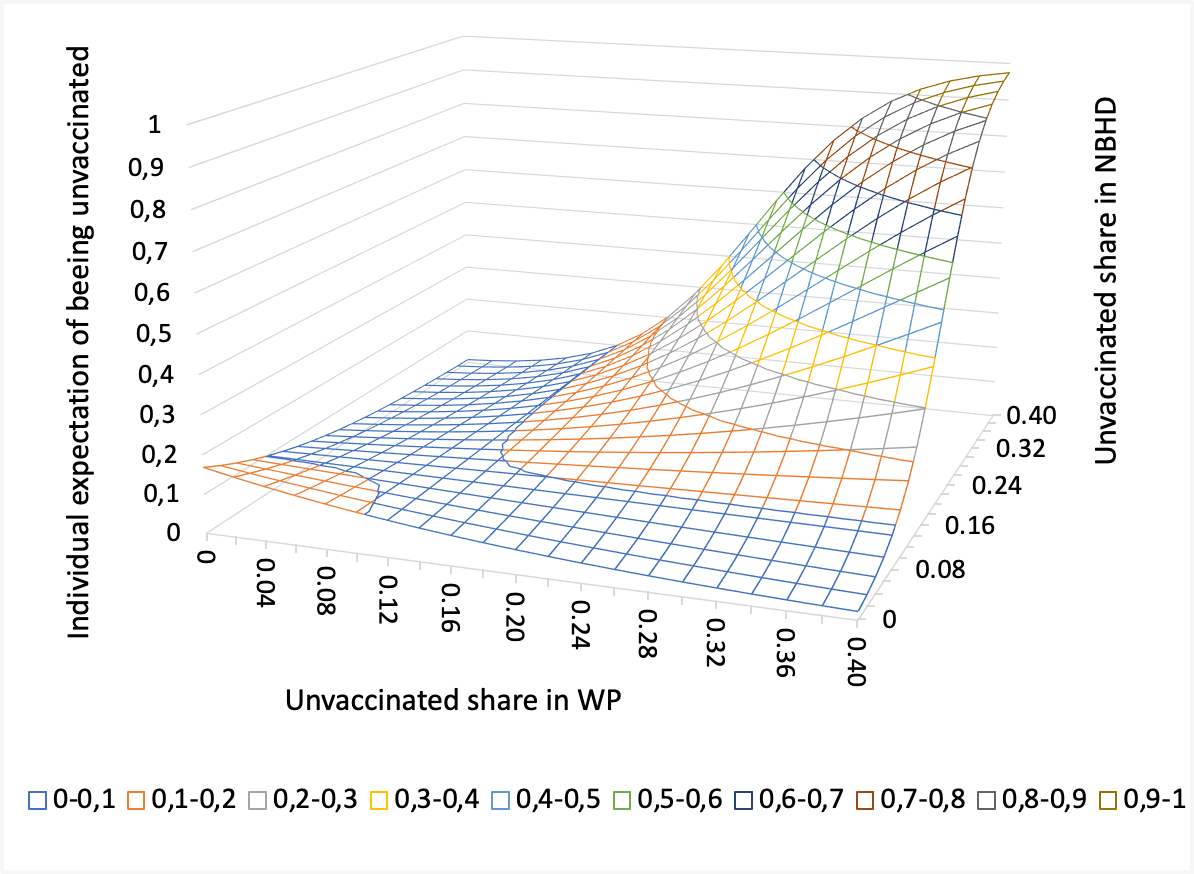 |
| --- | --- |
| High education | Low education |
| 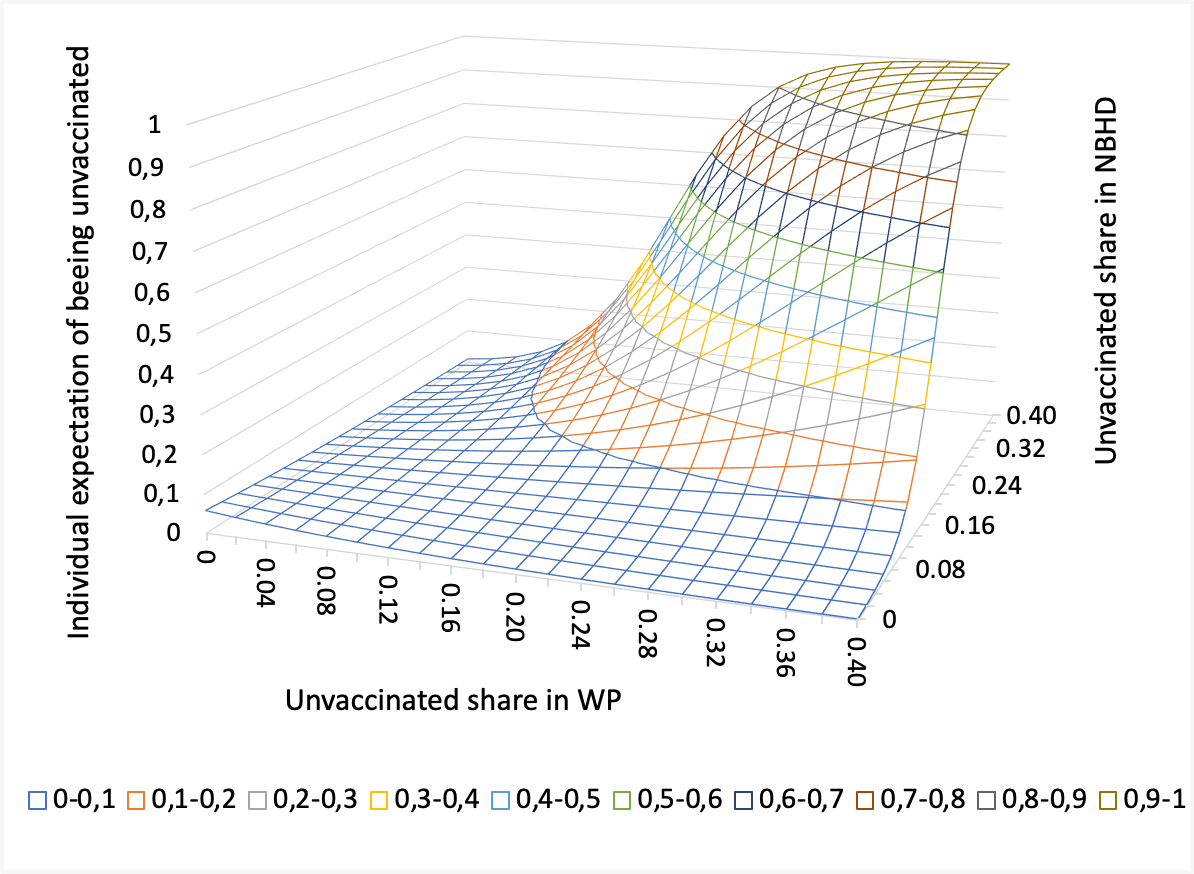 | 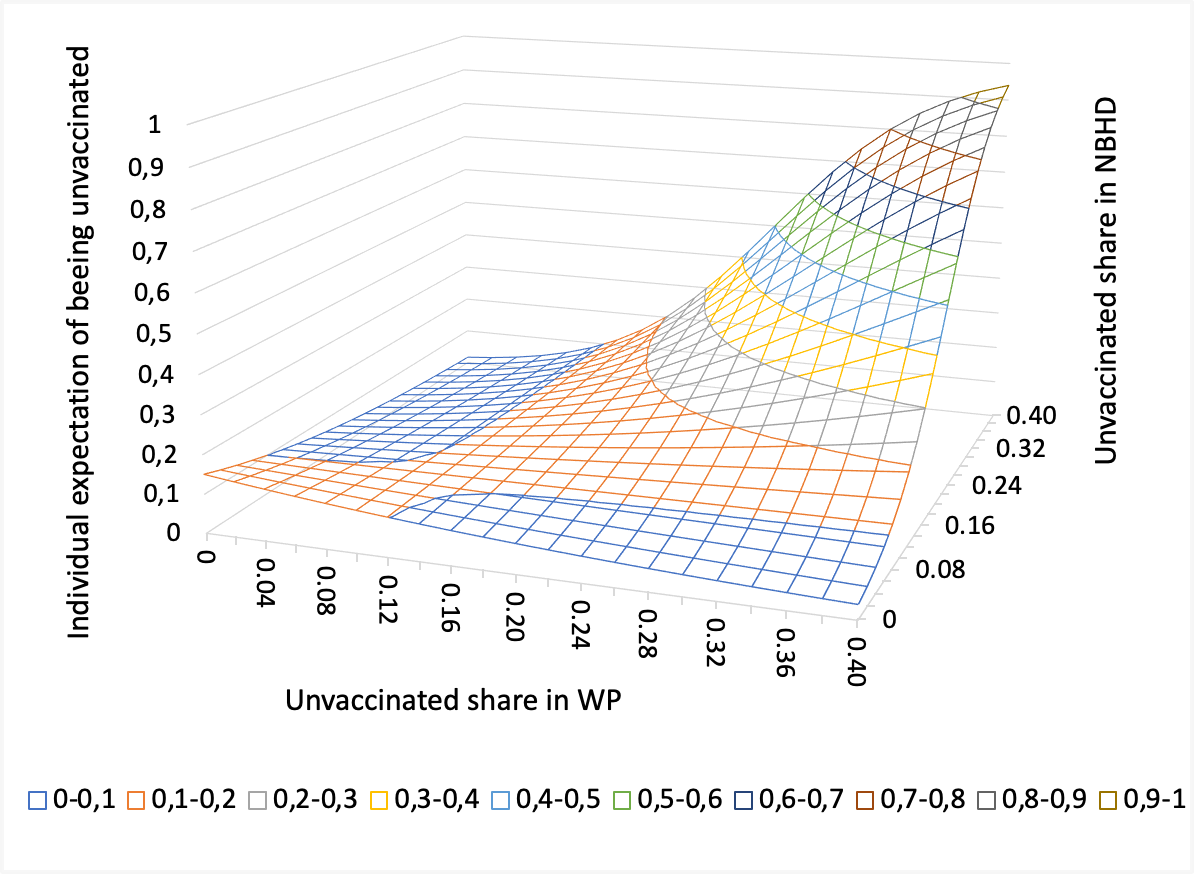 |
| High skill | Low skill |

**Probability surfaces of the individual expectation of being unvaccinated in relation to the unvaccinated shares in the workplace and the neighborhood (Different education levels and skill levels)**

1. North America and Oceania are grouped together due to the relatively low number of foreign-born individuals with origin in these regions, as well as the sharing of similar cultural backgrounds. [↑](#footnote-ref-1)
2. North America and Oceania are grouped together due to the relatively low number of foreign-born individuals with origin in these regions, as well as the sharing of similar cultural backgrounds. [↑](#footnote-ref-2)
3. In Sweden, households are classified as crowded if there are less rooms than household members, with the exception that married/co-habiting adults are assumed to share a bedroom. The number of rooms do not include a kitchen and a living room. Hence, single individuals living in one-room apartments are classified as crowded according to Swedish standards. [↑](#footnote-ref-3)
